# Supplementary material for: Causal Relationship between Aging and Anorexia Nervosa: A White-Matter-Microstructure-Mediated Mendelian Randomization Analysis
Source: Biomedicines. 2024 Aug 16;12(8):1874. doi: 10.3390/biomedicines12081874 (PMC11351342; doi:10.3390/biomedicines12081874)
Supplement: Supplementary file 1 [file biomedicines-12-01874-s001.zip › Supplementary Figure/Figure S8/Leaveoneout plot of MR analysis for TL to ubm-b-1983.pdf]

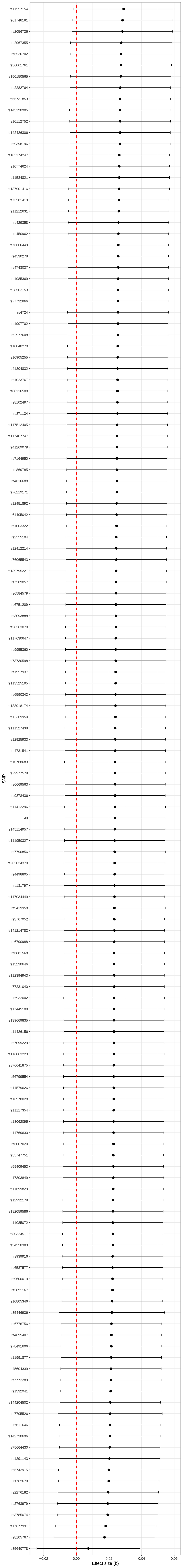
rs11557154
rs61748181
rs2056726
rs2967355
rs6536702
rs56061761
rs150150565
rs2282764
rs66731853
rs143190905
rs10112752
rs142426306
rs9398196
rs185174247
rs10774624
rs11584821
rs137901416
rs73581419
rs11212631
rs429358
rs450962
rs76664449
rs4530278
rs4743037
rs1985369
rs28502153
rs77732866
rs4724
rs1907702
rs2977608
rs10840270
rs10905255
rs41304832
rs1023767
rs80116508
rs8102497
rs871134
rs117512405
rs117407747
rs41269079
rs7164950
rs869785
rs4616688
rs76219171
rs12451892
rs61405042
rs1003322
rs2555104
rs12412214
rs76065543
rs139795227
rs7209057
rs6584579
rs6751209
rs3093888
rs28363070
rs117630647
rs9955360
rs73730598
rs1957937
rs113525195
rs6590343
rs188918174
rs12369950
rs111527438
rs12925933
rs4731541
rs10768683
rs79977579
rs6669563
rs9878436
rs11412296
All
rs145114957
rs111950327
rs7790856
rs202034370
rs4498805
rs131797
rs117034449
rs9419958
rs3767952
rs141214782
rs6790988
rs6881568
rs13230646
rs112394943
rs77231040
rs932002
rs17445108
rs139669835
rs11426156
rs7099229
rs116863223
rs376641875
rs56799554
rs11579626
rs16978028
rs11117354
rs13062095
rs11769630
rs6007020
rs55747751
rs59409453
rs17803849
rs11699829
rs12932179
rs182059586
rs11085072
rs80324517
rs34550383
rs939916
rs6587577
rs9600019
rs3891167
rs10805346
rs35446936
rs6776756
rs4695407
rs78491606
rs11991877
rs45604339
rs7772289
rs1332941
rs144204502
rs7705526
rs611646
rs142730696
rs75664430
rs1291143
rs55742915
rs762679
rs2276182
rs2763979
rs3785074
rs17677991
rs8105767
rs35640778
SNP
-0.02
0.00
0.02
0.04
0.06
Effect size (b)
